# Efficacy and safety of a crystalline lactulose preparation (SK-1202) in Japanese patients with chronic constipation: a randomized, double-blind, placebo-controlled, dose-finding study

**DOI:** 10.1007/s00535-018-01545-7

**Published:** 2019-01-14

**Authors:** Kunio Kasugai, Hisakazu Iwai, Noboru Kuboyama, Aya Yoshikawa, Shin Fukudo

**Affiliations:** 1 Division of Gastroenterology, Department of Internal Medicine, Aichi Medical University School of Medicine, 1-1 Yazakokarimata, Nagakute, Aichi 480-1195 Japan; 2 Sanwa Kagaku Kenkyusho Co., Ltd., Nagoya, Japan; 3 Department of Behavioral Medicine, Tohoku University Graduate School of Medicine, Sendai, Japan

**Keywords:** SK-1202, Lactulose, Japanese adult, Chronic constipation, Randomized controlled trial

## Abstract

**Background:**

Clinical evidence of lactulose for chronic constipation in Japan was lacking. We performed a randomized, double-blind, placebo-controlled, dose-finding study in Japanese patients with chronic constipation to estimate the optimal clinical dose of lactulose.

**Methods:**

Overall, 250 patients were randomized to receive SK-1202 (13, 26, or 39 g/day, as crystalline lactulose dosage) or placebo twice daily (morning and evening) orally for 2 weeks. The primary endpoint was the change from baseline frequency of spontaneous bowel movements (SBMs) at Week 1. The secondary endpoints included the change from baseline of SBMs at Week 2, percentage of patients experiencing SBM within 24 and/or 48 h of the initial dose, stool consistency, and constipation severity, and adverse events were also evaluated.

**Results:**

The 26 and 39 g/day of SK-1202 induced significantly and dose-dependently more increase in SBM at Week 1 than placebo (*p* = 0.003, *p* < 0.001). These groups also showed significant improvements in the secondary endpoints. There were no significant differences in the incidence of adverse drug reactions (ADRs) between the placebo and SK-1202 groups. Gastrointestinal disorder was the most common ADR, and diarrhea developed in 6 patients (9.7%) treated with 39 g/day; however, the symptoms were mild in severity and resolved after follow-up, dose reduction, or dose suspension. SK-1202 was generally well tolerated up to 39 g/day.

**Conclusion:**

Our results suggest that SK-1202 is useful in Japanese patients with chronic constipation, and optimal dose of SK-1202 is 26 g/day.

**Electronic supplementary material:**

The online version of this article (10.1007/s00535-018-01545-7) contains supplementary material, which is available to authorized users.

## Introduction

Lactulose is a synthetic disaccharide composed of fructose and galactose. Orally administered lactulose reaches the lower gastrointestinal tract unchanged, where lactulose increases the retention of water and electrolytes by its osmotic effect. Lactulose is broken down by enteric bacteria into organic acids (e.g., lactic and acetic acids) that stimulate bowel motility [1, 2]. Lactulose has been used in many countries for many years as a laxative not only for children but also for adults with doses ranging from 10 to 40 g/day and is recommended as a useful laxative in various therapeutic guidelines, including the “World Gastroenterology Organisation Global Guideline” [3], the “Guidance for the Prevention and Management of Constipation in Adults” [4] by the National Health Service, and the “American Gastroenterological Association Technical Review on Constipation” [5]. Moreover, in Japan, the “Evidence-based clinical practice guideline for chronic constipation 2017” strongly recommended to use osmotic laxatives including lactulose with high level (A) of evidence [6]. Actually lactulose preparations, such as Monilac^®^ (syrup/powder), have been marketed in Japan since 1979 for the treatment of pediatric constipation and to induce bowel movement/flatus expulsion after gynecological surgery. However, there was neither a randomized, placebo-controlled, double-blind, parallel-group study for the treatment of adult patients with chronic constipation nor approval of lactulose indicating for chronic constipation in Japan.

We performed a randomized, placebo-controlled, double-blind, parallel-group study of the SK-1202 formulation containing crystalline lactulose as an active ingredient in Japanese adult patients with chronic constipation to evaluate the efficacy and safety of the drug administered twice daily for 2 weeks and to determine the optimal dose for Japanese adult patients.

## Methods

### Patients

Japanese adult patients of either sex, aged ≥ 20 to < 75 years, experiencing chronic constipation as defined by the functional constipation without excluding irritable bowel syndrome with constipation (IBS-C) in the Rome III diagnostic criteria [7], were eligible to participate in this study, if they had provided written informed consent after receiving a full explanation of the study’s purpose, and met all of the inclusion criteria and none of the exclusion criteria, described below.

Main inclusion criteria were patients had to have fewer than 3 spontaneous bowel movements (SBMs) per week on average before the start of screening assessment and during the 2-week observation period, and to have at least 1 SBM-related symptom mentioned below from 1 to 3, which had lasted for at least 6 months.

1. Lumpy or hard stools in at least 25% of defecations.
2. Sensation of incomplete evacuation for at least 25% of defecations.
3. Straining during at least 25% of defecations.

Main exclusion criteria were as follows:

1. Patients with organic constipation;
2. Patients with megacolon or megarectum;
3. Patients with intestinal pseudo-obstruction;
4. Patients who underwent gastrointestinal surgery or an abdominal surgery within 1 year prior to the start of screening assessment, or patients with a history of gastrointestinal resection;
5. Patients with serious renal or hepatic disorders;
6. Patients who have received lactulose; and
7. Patients with galactosemia.

We did not exclude IBS-C patients who had recurrent abdominal pain or discomfort for at least 3 days per month in the last 3 months [7] from patients with chronic constipation. This strategy is supported by the Rome IV criteria as a newer concept of functional gastrointestinal disorders [8]. In the Rome IV criteria, functional constipation and IBS-C were conceptualized as spectrum disorders of the lower gastrointestinal tract [8]. This design was also supported by the evidence-based clinical practice guideline for chronic constipation 2017 in Japan [6].

### Drugs

SK-1202 consisted of a jelly preparation containing 6.5 g of crystalline lactulose (with a purity of ≥ 97%, hereinafter referred to as “lactulose”) in one sachet (Sanwa Kagaku Kenkyusho Co., Ltd., Nagoya, Japan), and the placebo was a jelly preparation that was indistinguishable in appearance and taste from SK-1202 but contained no lactulose (Sanwa Kagaku Kenkyusho Co., Ltd.).

### Study design

**Fig. 1:**
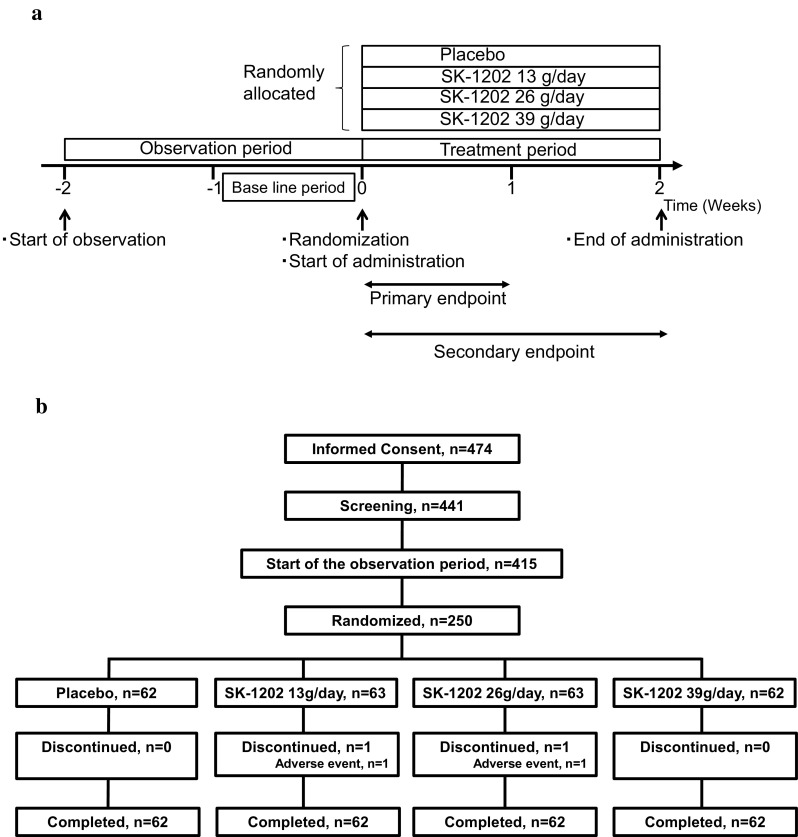
**a** Design of the study. **b** Flow chart of patient enrollment, allocation, and study implementation

This was a multicenter, randomized, placebo-controlled, double-blind, parallel-group study conducted in the 19 medical institutions specialized in gastroenterology in Japan between August 2013 and February 2014 (no. of subjects with informed consent: from 17 to 31 subjects/institution). The study design is presented in Fig. 1a. The doses of SK-1202 tested in this study were set at 0 (placebo), 13, 26, and 39 g lactulose/day based on the doses approved overseas for the treatment of adult constipation, ranging from 10 to 40 g/day [9]. Patients assigned to the 13 g/day group received 1 sachet of SK-1202 and 2 sachets of placebo; those to the 26 g/day group, 2 sachets of SK-1202 and 1 sachet of placebo; those to the 39 g/day group, 3 sachets of SK-1202; and those to the placebo group, 3 sachets of placebo twice daily (morning and evening) orally for 2 weeks. The dosing frequency for Week 2 could be reduced to once daily depending on the patient’s symptoms, at the discretion of the investigator/sub-investigator. Returning to the originally assigned dose after dose reduction was not permitted.

The sample size in each group was determined as follows: assuming that the change from baseline SBM frequency was increased in the placebo group by 0.98/week in the 26 g/day group and by 1.5/week in the 39 g/day group, with a standard deviation of 1.8/week for both groups, based on the data from the overseas study [10, 11], 55 patients in each group would be required to detect significant increases from the placebo group in SBM frequency in the 26 g/day group and the 39 g/day group with a power of 80% at a significance level of 0.05. Taking into account study discontinuations/dropouts, the sample size necessary for each group was determined to be 62.

If rescue therapy was required for 24 h before or 48 h after the first dose of the study drug, or if the investigator/sub-investigator considered that the continued participation in the study would be difficult due to adverse events, the patient was withdrawn from the study. If no complete defecation occurred for 3 consecutive days in the observation period or in the treatment period, the use of standard rescue therapy with bisacodyl suppository or glycerin enema was provided.

This study was reviewed and approved by the central institutional review board (Mano medical clinic, Approved number: 20130808) and conducted in accordance with the ethical principles that have their origins in the Declaration of Helsinki and in adherence to GCP, the applicable regulatory requirements, and the protocol. This study was registered with Japan Pharmaceutical Information Center, number Japic CTI-132219.

### Endpoints

The primary efficacy endpoint was the change from baseline SBM frequency at Week 1 (the mean SBM frequency at Week 1 of the treatment period—the mean SBM frequency at the baseline period (i.e., the last week of the 2-week observation period)) [12]. The weekly number of SBMs was calculated by the following equation. (Number of spontaneous defecation in evaluable days/number of days that can be evaluated) × 7. When patients recorded rescue medication use, the subsequent 24 h were excluded from calculations of SBM frequency. The secondary efficacy endpoints included the change from baseline SBM frequency at Week 2 (the mean SBM frequency at Week 2 of the treatment period—the mean SBM frequency at the baseline period), the percentage of patients experiencing SBMs within 24 h or 48 h after the first dose of the study drug, and the time to the first SBM from the first dose of the study drug. Other assessments at each week of treatment included the stool consistency score [13] and its change, the global assessment score of constipation severity, and the Irritable Bowel Syndrome Quality of Life (IBS-QOL) [14] score.

Stool consistency was categorized into the following 7 types according to the Bristol Stool Form Scale (BSFS) [13]: type 1, separate hard lumps, like nuts (hard to pass); type 2, sausage-shaped but lumpy; type 3, like a sausage but with cracks on its surface; type 4, like a sausage or snake, smooth and soft; type 5, soft blobs with clear cut edges (passed easily); type 6, fluffy pieces with ragged edges, a mushy stool; and type 7, watery, no solid pieces, entirely liquid. The global assessment score of constipation severity was categorized into the following 5 levels: 0, none (symptoms absent); 1, mild (slight symptoms present); 3, moderate (constipated, but the symptoms were not severe); 3, severe (severely constipated, with difficulty in defecating or with faint sensation of the need to defecate; and 4, very severe (extremely constipated, with few defecations or with little sensation of the need to defecate). IBS-QOL was assessed using the Japanese version of the IBS-QOL (acute) [14] and the results were calculated using subscales.

The safety endpoints were adverse events, vital signs, resting 12-lead electrocardiogram, and laboratory test results. The clinical investigators judged the medical event unfavorable to the patient as an adverse event. For example, if the clinical investigators considered that diarrhea caused by softening of feces was preferable for a patient, it was not regarded as an adverse event. Adverse events were coded using the Japanese translation of the Medical Dictionary for Regulatory Activities (MedDRA/J), ver. 16.1. Adverse events for which causal relationship with the study drug could not be ruled out were defined as adverse drug reactions. These safety endpoints, except adverse events, were assessed before the first dose of the study drug and at the completion (or discontinuation) of the 2-week study treatment.

### Statistical analyses

Efficacy analyses were performed on the full analysis set composed of patients who had at least 1 day with evaluable on-treatment efficacy data. Safety analyses were performed on the safety population composed of patients who received at least 1 dose of the study drug. The change from baseline SBM frequency at Week 1, the primary endpoint, was summarized group-wise using descriptive statistics, and pairwise comparisons between each SK-1202-treated group and the placebo group were performed from the highest dose group to the lowest dose group according to the closed testing procedure, using an analysis of covariance (ANCOVA) model including the baseline SBM frequency as a covariate. The dose–response relationship was assessed using the maximum contrast method.

Among the secondary endpoints, the change from baseline SBM frequency at Week 2, the stool consistency score, and the IBS-QOL score were summarized using descriptive statistics and an ANCOVA including the frequency/score at baseline as a covariate. Number and percentages of patients experiencing SBMs within 24 h and 48 h after the first dose of the study drug were summarized by groups to compare each SK-1202-treated group with the placebo group using the Fisher’s exact probability test. The global assessment score of constipation severity was summarized by week and group to compare each SK-1202-treated group with the placebo group using the Wilcoxon rank sum test. Time to the first SBM from the first dose of the study drug was analyzed using Kaplan–Meier curves and compared between each SK-1202-treated group and the placebo group by means of the generalized Wilcoxon test. The median time to the first SBM in each group was determined.

The number of patients experiencing adverse events and adverse drug reactions and their respective incidence rates were calculated for each group to compare each SK-1202-treated group and the placebo group using the Fisher’s exact probability test.

A 15% significance level for two-sided tests was used for demographic and other baseline data, a one-sided significance level was set to 2.5% for the contrast test used to assess the dose–response relationship, and a 5% significance level for two-sided tests was set for other statistical tests, unless otherwise specified.

## Results

### Patients analyzed and characteristics

The disposition of the patients evaluated is presented in Fig. 1b. Excluding 26 screen failures and 165 inclusion/exclusion criteria violations, 250 patients were randomly assigned to the placebo group (62 patients), the SK-1202 13 g/day group (63 patients), the 26 g/day group (63 patients), or the 39 g/day group (62 patients). During the study, 2 patients were withdrawn from the study due to adverse drug reactions (mild abdominal distension in 1 patient in the 13 g/day group and moderate urticaria in 1 patient in the 26 g/day group). Overall, 248 patients completed the study treatment.

The characteristics of the patients participating in the study are shown in Table 1. Each group had more female patients (77.8–87.1%), with a mean age of 39.4–43.1 years and a mean body mass index of 20.94–21.99 kg/m^2^. The SBM frequency in the baseline period in each group ranged between 1.47 and 1.57/week, with no significant difference among the groups.

**Table 1:** Patient characteristics and baseline (safety population)

Characteristics	Placebo	SK-1202			*p* value*
		13 g/day	26 g/day	39 g/day	
	*n* = 62	*n* = 63	*n* = 63	*n* = 62	
Sex, *n* (%)					
Male	9 (14.5)	9 (14.3)	14 (22.2)	8 (12.9)	0.505^a^
Female	53 (85.5)	54 (85.7)	49 (77.8)	54 (87.1)	
Age, years					
Mean ± SD	42.3 ± 11.6	39.4 ± 12.3	42.8 ± 12.7	43.1 ± 11.0	0.286^b^
BMI, kg/m^2^					
Mean ± SD	20.94 ± 3.19	21.47 ± 2.91	21.99 ± 3.99	21.55 ± 3.11	0.369^b^
Duration of constipation (months)					
Mean ± SD	217.9 ± 143.7	198.5 ± 145.9	192.4 ± 135.9	187.0 ± 139.9	0.638^b^
History of treatment for constipation, *n* (%)					
No	29 (46.8)	42 (66.7)	35 (55.6)	31 (50.0)	0.122^a^
Yes	33 (53.2)	21 (33.3)	28 (44.4)	31 (50.0)	
Constipation-predominant IBS, *n* (%)					
No	55 (88.7)	59 (93.7)	60 (95.2)	56 (90.3)	0.500^a^
Yes	7 (11.3)	4 (6.3)	3 (4.8)	6 (9.7)	
The frequency of SBMs at baseline, time/week					
Mean ± SD	1.47 ± 0.79	1.50 ± 0.74	1.57 ± 0.61	1.55 ± 0.64	0.831^b^

*SD* standard deviation, *BMI* body mass index, *IBS* irritable bowel syndrome, *SBM* spontaneous bowel movement

*The two-sided level of significance is 15%

^a^Fisher’s Exact Test

^b^One-way ANOVA

There was a significant difference in the presence/absence of previous constipation treatments among the groups (Fisher’s exact probability test, *p* = 0.122). However, no association between the presence/absence of previous constipation treatments and the change from baseline SBM frequency at Week 1, the primary endpoint, was observed. Therefore, the difference in the previous constipation treatments was believed to have no effect on the primary endpoint. In 26 g/day and 39 g/day groups, there was a significant difference from control group in change from the baseline SBM in the patients with the presence or absence of treatment history of constipation. It is considered that treatment with SK-1202 improved constipation in comparison with previous treatment.

### Efficacy

**Fig. 2:**
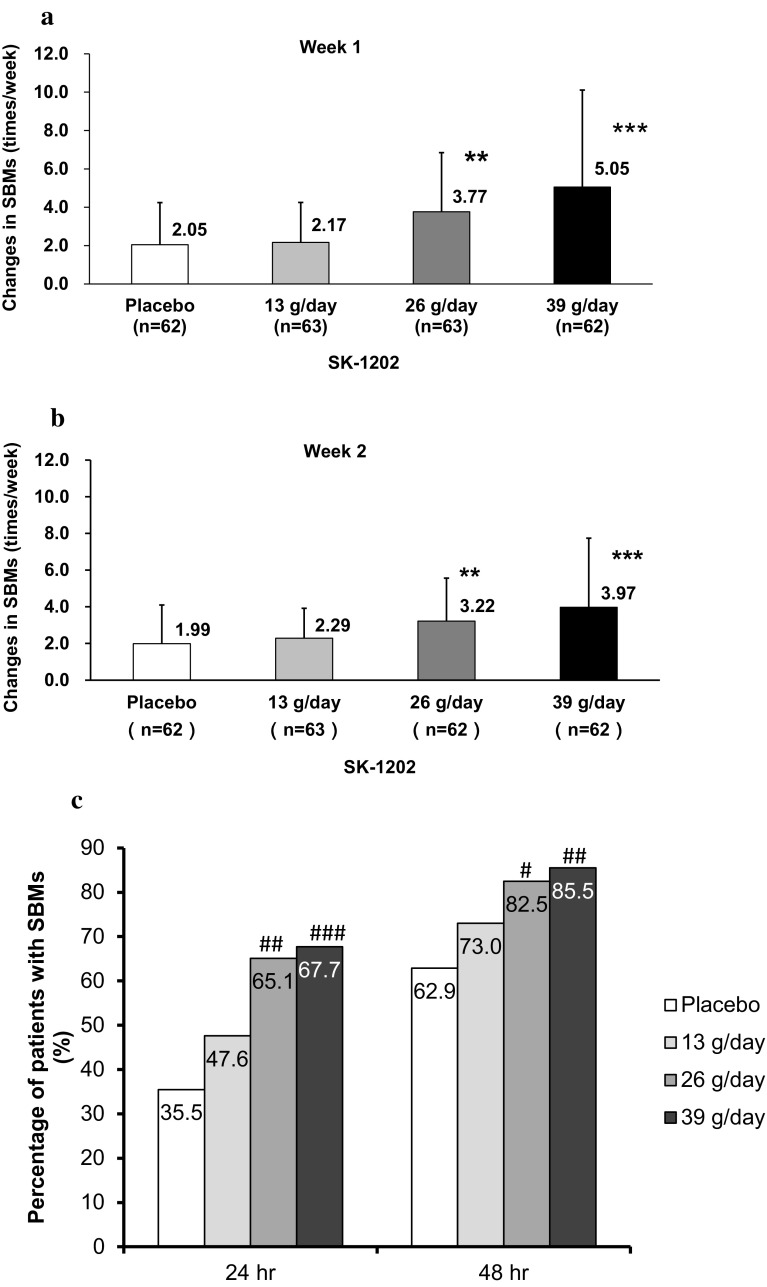
Dose-related effects of SK-1202 on spontaneous bowel movements (SBMs) in patients with chronic constipation. **a** Changes in SBMs from baseline at Week 1. **b** Change in SBMs from baseline at Week 2. **c** Percentage of patients who had initial SBM within 24 or 48 h after initiation of SK-1202 treatment. The columns and bars represent mean ± standard deviation in **a** and **b**. The columns and numbers represent mean of percentage of patients in **c**. Significant differences in changes in SBMs from baseline based on ANCOVA with average weekly SBMs at baseline as covariate: *p < 0.05, **p < 0.01, ***p < 0.001 vs. placebo. Significant differences in the percentage of patients with initial SBM based on Fisher’s Exact Test, ^#^*p* < 0.05, ^##^*p* < 0.01, ^###^*p* < 0.001 vs placebo

The mean change from baseline SBM frequency at Week 1, the primary endpoint, was 2.17/week in the 13 g/day group, 3.77/week in the 26 g/day group, and 5.05/week in the 39 g/day group; whereas, it was 2.05/week in the placebo group. An ANCOVA showed that the increases in the SBM frequency in the 26 g/day group and in the 39 g/day group were significantly higher than in the placebo group (*p* = 0.003 and *p* < 0.001, respectively) (Fig. 2a). In the assessment of the dose–response relationship, a contrast test using contrast coefficient (− 3, − 3, 1, 5) for treatment groups (placebo, SK-1202 13 g/day, 26 g/day, and 39 g/day) provided the maximum test statistic (*t* = 5.877, *p* < 0.001). It indicated that the dose–response relationship of SK-1202 for the primary endpoint was apparent at 26 g/kg or higher dose; whereas, the response plateaued between the placebo group and the 13 g/day group. There was no major difference in change of SBM frequency between all the patients and the patients without rescue medication (data not shown).

Similar to the primary endpoint, the change from baseline SBM frequency at Week 2 increased as the dose increased, and significant differences from the placebo group were found for the 26 g/day group and the 39 g/day group (*p* = 0.007 and *p* < 0.001, respectively) (Fig. 2b). The percentage of patients experiencing the first SBM within 24 h after the first dose of the study drug rose as the dose increased, and it was significantly higher in the 26 g/day and in the 39 g/day groups (65.1% and 67.7%, respectively) than in the placebo group (*p* = 0.001 and *p* < 0.001, respectively). Similarly, the percentage of patients experiencing the first SBM within 48 h after the first dose of the study drug rose as the dose increased, and it was significantly higher in the 26 g/day group and in the 39 g/day group (82.5% and 85.5%, respectively) than in the placebo group (*p* = 0.022 and *p* = 0.007, respectively) (Fig. 2c).

**Fig. 3:**
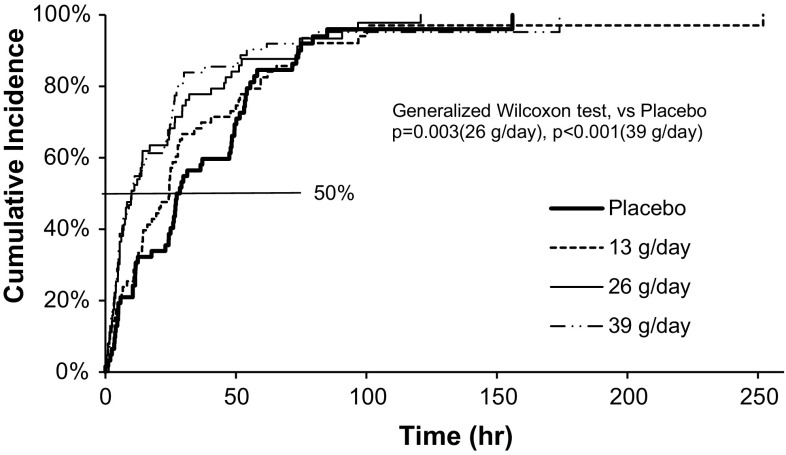
Kaplan–Meier curve for time (hours) to initial spontaneous bowel movement incidence in each group. Time (hour) represents required number of hours to induce an initial spontaneous bowel movement (SBM) after the first SK-1202 dose. Cumulative incidence represents ratio of patients with initial SBM

The time to the first SBM from the first dose of the study drug is presented in Fig. 3.

The time to the first SBM was significantly shorter in the 26 g/day and in the 39 g/day groups than in the placebo group (*p* = 0.003 and *p* < 0.001, respectively). The median time to the first SBM was 24.50 h [95% confidence interval (CI), 14.33–27.77] in the 13 g/day group, 10.00 h (95% CI 6.08–17.00) in the 26 g/day group, and 10.33 h (95% CI 5.45–22.58) in the 39 g/day group, as compared to 27.98 h (95% CI 24.33–48.00) in the placebo group; thus, it was significantly shorter in the 26 g/day group and the 39 g/day group than in the placebo group (Fig. 3).

**Fig. 4:**
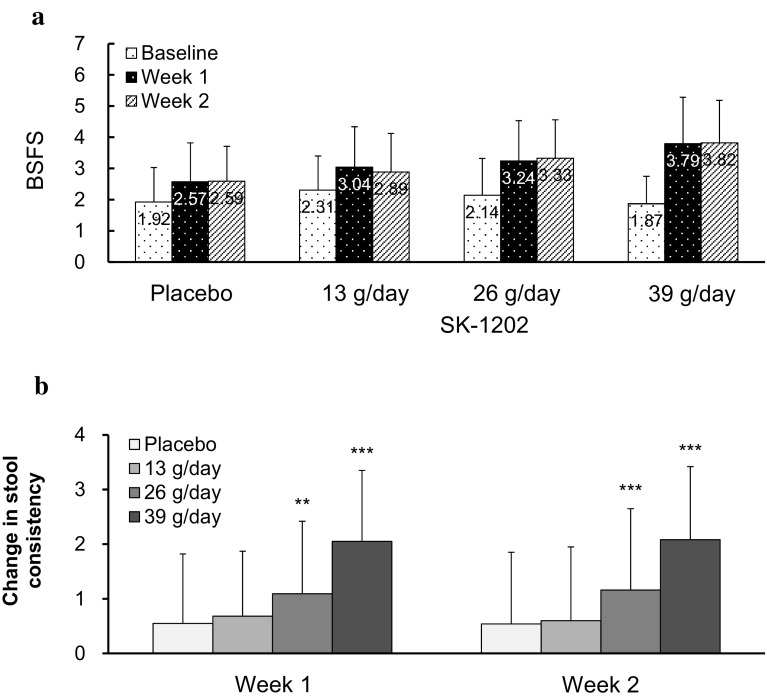
**a** Serial changes in BSFS in each group. **b** Changes in stool consistency from baseline by BSFS. *BSFS* Bristol Stool Form Scale. **a** The stool consistency was based on BSFS. The columns and numbers represent the mean BSFS, and the bars represent the standard deviation. **b** Difference in the change in BSFS was analyzed with the use of ANCOVA model with mean weekly score at baseline as covariate **p < 0.01, ***p < 0.001 vs placebo. The markers and bars represent mean and standard deviation

BSFS was close to ideal stool consistency of “3–5”in the 26/day and in the 39 g/day groups (Fig. 4a). And the changes in the stool consistency from the baseline scale at Week 1 in the 26 g/day group and the 39 g/day group significantly improved compared to the placebo group (*p* < 0.02 and *p* < 0.001, respectively). The significant improvements observed in the 26 g/day and the 39 g/day groups persisted up to Week 2 (*p* < 0.001 for both groups, Fig. 4b).

**Fig. 5:**
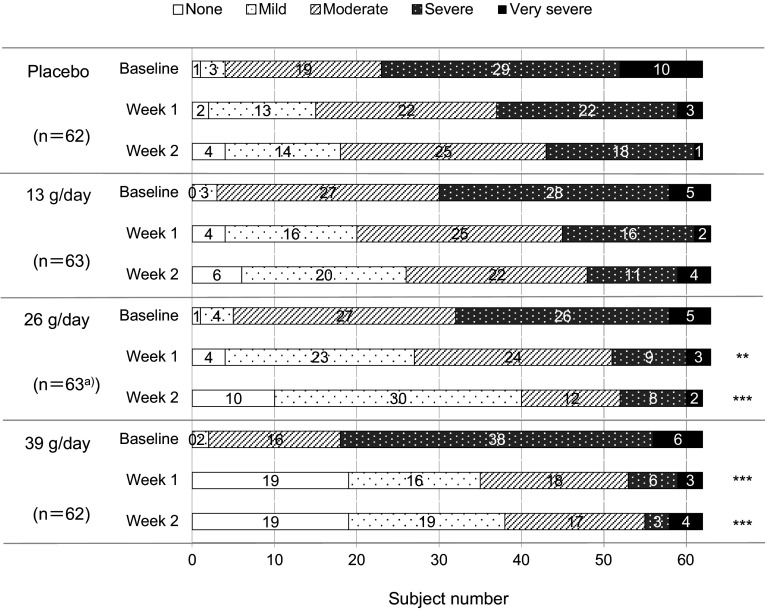
Serial changes from baseline in the number of patients with constipation stratified by severity. Top, each square represents severity of constipation. **a** The data for one patient were missing in week 2. **p < 0.01, ***p < 0.001 vs. placebo (by Wilcoxon rank sum test)

The global assessment scores of constipation severity are shown in Fig. 5. There was no statistically significant difference in the severity of constipation between placebo group and 13 g/day, 26 g/day and 39 g/day groups at baseline period. The proportion of patients with “symptoms absent” was 1 of 62 patients in the placebo group, 0 of 63 patients in the 13 g/day group, 1 of 63 patients in the 26 g/day group, and 0 of 62 patients in the 39 g/day group in the baseline period, which increased dose dependently to 4 of 62, 6 of 63, 10 of 63, and 19 of 62 patients, respectively, at Week 2. The global assessment score of constipation severity at Weeks 1 and 2 in the 26 g/day group and in the 39 g/day group improved more significantly than in the placebo group (*p* < 0.01 and *p* < 0.001 at Week 1, *p* < 0.001 for both groups at Week 2).

The change from baseline IBS-QOL-J, acute score at Week 2 was analyzed using an ANCOVA including the score at baseline as a covariate. The differences in least squares mean relative to the placebo group revealed a trend toward improvement in the subscales of healthy worry and food avoidance in the 26 g/day group and significant improvements in the subscales of body image, healthy worry, food avoidance, and overall (*p* = 0.003, *p* < 0.001, *p* = 0.022, and *p* = 0.028, respectively) in the 39 g/day group (Supplementary Fig. S1).

### Safety

No deaths were reported, and no patients experienced any other serious adverse events or adverse drug reactions. In addition, no patients experienced any severe adverse events. Study treatment was discontinued in 1 patient due to adverse drug reactions in the 13 g/day group (1.6%, abdominal distension [mild, resolved]) and in 1 patient in the 26 g/day group (1.6%, urticaria [moderate, resolved]), with no discontinuations due to adverse drug reactions in the placebo group and the 39 g/day group. The numbers of patients with adverse drug reactions and their respective incidence rates are shown in Table 2. There were no significant differences between the SK-1202-treated groups and the placebo group. The adverse drug reactions reported in ≥ 2% of patients in any of the SK-1202-treated groups were diarrhea, abdominal pain, abdominal distension, and gastrointestinal sounds abnormal, all of which were classified under the System Organ Class (SOC) of “gastrointestinal disorders”. In the 39 g/day group, a total of 13 patients (21.0%) experienced these adverse drug reactions, and in particular, diarrhea was reported in more patients [6 patients (9.7%)]. All these adverse drug reactions were mild in severity and resolved or improved with no treatment for the reactions, by study treatment suspension, or dose reduction.

**Table 2:** Summary of adverse drug reactions in each group

	Placebo, *n* (%)	SK-1202, *n* (%)		
		13 g/day	26 g/day	39 g/day
	*n* = 62	*n* = 63	*n* = 63	*n* = 62
At least one ADR	6 (9.7)	2 (3.2)	4 (6.3)	13 (21.0)
Gastrointestinal disorders^a^				
Abdominal distension	0 (0.0)	1 (1.6)	1 (1.6)	2 (3.2)
Abdominal pain	2 (3.2)	0 (0.0)	1 (1.6)	2 (3.2)
Diarrhea	0 (0.0)	0 (0.0)	0 (0.0)	6 (9.7)
Gastrointestinal sounds abnormal	0 (0.0)	0 (0.0)	1 (1.6)	2 (3.2)
Investigations^a^				
Protein total decreased	2 (3.2)	0 (0.0)	0 (0.0)	0 (0.0)

*ADR* adverse drug reaction

^a^Adverse drug reactions reported by more than 2% of subjects in any group are shown

## Discussion

This is the first study that lactulose is actually effective without any serious adverse events for patients with chronic constipation in Japan. Lactulose is recommended as a useful laxative by different guidelines for the treatment of constipation [3–6] and is widely used overseas. In Japan, however, it has not been approved for the treatment of adult constipation. Bass et al. administered lactulose at a dose of 40 g/day for 1 week to evaluate its effects on stool frequency [11]. The stool frequency in the lactulose group increased from 1.6/week at baseline to 4.5/week at Week 1; while it, in the placebo group, increased from 1.4/week at baseline to 2.8/week at Week 1. The adverse events reported in the lactulose group were abdominal discomfort, nausea, severe diarrhea, and excessive flatulence. The usual adult dose of lactulose approved overseas for the treatment of chronic constipation is 10–40 g/day. We, therefore, performed the first randomized, placebo-controlled, double-blind, parallel-group study in Japanese adult patients with chronic constipation to evaluate the efficacy and safety and to determine the optimal dose of SK-1202, a lactulose preparation, which was administered at a dose of 13 g/day, 26 g/day or 39 g/day twice daily for 2 weeks.

The change from baseline SBM frequency at Week 1, the primary endpoint, was greater in the 26 g/day group and the 39 g/day group than in the placebo group, indicating a dose–response relationship between the 26 g/day and 39 g/day. Our dose of lactulose with 39 g/day was almost comparable dose in the previous study [11]. There was no major difference in change of SBM frequency between all the patients and the patients without rescue medication. Therefore, the usage of rescue medication did not affect the results. These results supported the efficacy of SK-1202 administered in doses of 26 g/day or higher in treating adult constipation. The change from baseline SBM frequency at Week 2 was also greater in the 26 g/day and 39 g/day groups than in the placebo group. The changes in the 26 g/day group were similar between Week 1 and Week 2, but in the 39 g/day group, the change at Week 1 was higher than it at Week 2. In the 39 g/day group, some patients who experienced diarrhea at Week 1 reduced daily dose at Week 2. Therefore, we considered that the change from baseline SBM frequency in 39 g/day at Week 1 might be caused by dosing of the surplus amount of lactulose to the subjects.

The percentage of patients experiencing SBMs within 24 h and 48 h after the first dose of the study drug, the time to the first SBM from the first dose, global assessment score of constipation severity, and the change in stool consistency showed a more improvement in the 26 g/day group and in the 39 g/day group than in the placebo group. With regard to the safety profile of SK-1202, there were no differences in the number of patients or in the incidence rate of adverse drug reactions among the SK-1202-treated groups. In the 39 g/day group, the adverse drug reactions of diarrhea, gastrointestinal sounds abnormal, abdominal distension, and abdominal pain, all of which were classified under the SOC of “gastrointestinal disorders”, were frequently reported, and in particular, diarrhea occurred in more patients [6 patients (9.7%)]. The earlier randomized controlled trial of lactulose for elderly patients with chronic constipation reported that administration of 8–30 ml/day of 50% lactulose did not induce diarrhea [15]. In this study, all adverse drug reactions were mild, and resolved or improved with no treatment for the reactions, or by study treatment suspension or dose reduction. Five patients in the 39 g/day group required dose reduction due to diarrhea, gastrointestinal sounds abnormal, or other reasons, suggesting that the dose of 39 g/day may be supratherapeutic and should be reduced for some patients.

There have been several studies evaluating the efficacy and safety of stimulant laxatives in patients with chronic constipation as defined by the Rome III diagnostic criteria. The percentage of patients experiencing their first SBM within 24 h after the first dose of picosulfate was 69% [16], which was not largely different from the percentage obtained in the SK-1202 (65.1%). The mean time to the first SBM from the first dose of bisacodyl was 12 h [17], which was similar to the time observed in the SK-1202 (10 h). The incidence rate of adverse drug reactions, such as diarrhea, in patients receiving 4-week bisacodyl therapy was 63.6% (157 of 247 patients); whereas, it was 6.3% in patients receiving 26 g/day of SK-1202 for 2 weeks in this study, suggesting that the drugs have different safety profiles.

At the time of beginning of this study, lubiprostone was the only agent for which the clinical effect and safety were proven with the recent design of randomized double-blind controlled trial in Japan [12]. Lubiprostone consists of new action mechanism and selectively activates the type 2 chloride channel, which increases fluid secretion in the intestinal apical cell membrane. On the other hand, lactulose reaches the lower gastrointestinal tract unchanged, where lactulose increases the retention of water and electrolytes by its osmotic effect. The changes from baseline SBM frequency at Weeks 1 and 2 in Japanese patients with chronic idiopathic constipation treated with the recommended clinical dose (48 μg/day) of lubiprostone, a secretagogue, were 3.66/week and 2.74/week, respectively [12]; whereas, the changes were 3.24/week and 3.33/week observed in the 26 g/day SK-1202-treated group. The percentage of patients experiencing SBMs within 24 h after the first dose of SK-1202 26 g/day group was 65.1% relative to 35.5% in the placebo group. This percentage was considered to be comparable with 58.1% relative to 30.6% in the placebo group as the percentage of patients experiencing SBMs within 24 h after the first dose of lubiprostone.

Abe et al. reported the safety of lubiprostone in 133 patients with chronic constipation (average age: 65 years old) who were given at 24 μg twice a day [18]. In their study, the incidence of experienced adverse drug reactions (ADRs) in the first 2 weeks after administration was 41.4% (55/133 patients), and nausea and diarrhea occurred at 24% and 17%, respectively. In our study with SK-1202, incidence of experienced ADRs was 6.3% in the 26 g/day group and 21.0% in the 39 g/day group. Nausea and diarrhea occurred only in the 39 g/day group, and incidence of nausea and diarrhea was 1.6% (1/62 patients) and 9.7% (6/62 patients), respectively. We consider the reason for this difference as follows. SK-1202 has osmotic effect in the lower gastrointestinal tract, and enhances bowel motility stimulated by organic acid to which lactulose is broken down by enterobacteria. On the other hand, lubiprostone has mechanism of action enhancing fluid secretion directly in the intestinal apical cell membrane. Therefore, the ADR of SK-1202 may be relatively less frequent than that of lubiprostone.

There were several limitations in this study. First, patients with IBS-C defined by the Rome III diagnostic criteria [7] were eligible for participation. However, the proportion of patients with IBS-C enrolled in the study with respect to the entire study population was low: 7 of 62 patients (11.3%) in the placebo group, 4 of 63 patients (6.3%) in the 13 g/day group, 3 of 63 patients (4.8%) in the 26 g/day group, and 6 of 62 patients (9.7%) in the 39 g/day group (Table 1). Therefore, the effects of SK-1202 on IBS-C could not be determined in this study and would require further investigation. Second, ratio of active compound: placebo was not 1:1 in this study. However, this is the first randomized controlled trial of lactulose in Japanese patients with chronic constipation. We consider that the design of this study is enough to support the Japanese evidence of lactulose as effective agents for chronic constipation as was previously and internationally meta-analyzed [19].

In conclusion, SK-1202, when administered twice daily for 2 weeks at a dose of 26 g/day or 39 g/day to Japanese adult patients with chronic constipation, was more effective than placebo, and a dose–response relationship was demonstrated between the 26 g/day and 39 g/day dosages. SK-1202 was generally well tolerated, except for the increased incidence of diarrhea in the 39 g/day group. The optimal dose of SK-1202 in the treatment of chronic constipation was, therefore, estimated to be 26 g/day. This dose falls within the range of the doses approved overseas for the treatment of adult constipation (10–40 g/day), and treatment with SK-1202 caused no serious or severe adverse drug reactions specific to Japanese patients in this study, indicating no ethnic difference in the efficacy and safety of lactulose. Based on these results, SK-1202 is expected to be a useful drug in the treatment of adult constipation, both for its efficacy and safety.

## Electronic supplementary material

Below is the link to the electronic supplementary material.

Supplementary material 1 (DOCX 87 kb)

## Abbreviations

ADRs: Adverse drug reactions
BSFS: Bristol Stool Form Scale
IBS-C: Irritable bowel syndrome with constipation
SBMs: Spontaneous bowel movements
SOC: System organ class
