# Supplementary material for: Efficacy and safety of a crystalline lactulose preparation (SK-1202) in Japanese patients with chronic constipation: a randomized, double-blind, placebo-controlled, dose-finding study
Source: J Gastroenterol. 2019 Jan 14;54(6):530–40. doi: 10.1007/s00535-018-01545-7 (PMC6536483; doi:10.1007/s00535-018-01545-7)
Supplement: Supplementary file 1 — Supplementary material 1 (DOCX 87 kb) [file 535_2018_1545_MOESM1_ESM.docx]

**Supplementary Fig. S1** The change from baseline on IBS-QOL score at Week 2.

Data are expressed as means
